# Detection of *MDM2* amplification by shallow whole genome sequencing of cell-free DNA of patients with dedifferentiated liposarcoma

**DOI:** 10.1371/journal.pone.0262272

**Published:** 2022-01-05

**Authors:** Joanna Przybyl, Lien Spans, Kristen Ganjoo, Nam Bui, David Mohler, Jeffrey Norton, George Poultsides, Maria Debiec-Rychter, Matt van de Rijn

**Affiliations:** 1 Department of Surgery, McGill University, Montreal, QC, Canada; 2 Cancer Research Program, The Research Institute of the McGill University Health Centre, Montreal, QC, Canada; 3 Department of Human Genetics, KU Leuven and University Hospitals Leuven, Leuven, Belgium; 4 Division of Medical Oncology, Department of Medicine, Stanford University, Stanford, CA, United States of America; 5 Department of Orthopaedic Surgery, Stanford University, Stanford, CA, United States of America; 6 Department of Surgery, Stanford University, Stanford, CA, United States of America; 7 Department of Pathology, Stanford University School of Medicine, Stanford, CA, United States of America; University of Texas M. D. Anderson Cancer Center, UNITED STATES

## Abstract

High-level amplification of *MDM2* and other genes in the 12q13–15 locus is a hallmark genetic feature of well-differentiated and dedifferentiated liposarcomas (WDLPS and DDLPS, respectively). Detection of this genomic aberration in plasma cell-free DNA may be a clinically useful assay for non-invasive distinction between these liposarcomas and other retroperitoneal tumors in differential diagnosis, and might be useful for the early detection of disease recurrence. In this study, we performed shallow whole genome sequencing of cell-free DNA extracted from 10 plasma samples from 3 patients with DDLPS and 1 patient with WDLPS. In addition, we studied 31 plasma samples from 11 patients with other types of soft tissue tumors. We detected *MDM2* amplification in cell-free DNA of 2 of 3 patients with DDLPS. By applying a genome-wide approach to the analysis of cell-free DNA, we also detected amplification of other genes that are known to be recurrently affected in DDLPS. Based on the analysis of one patient with DDLPS with longitudinal plasma samples available, we show that tracking *MDM2* amplification in cell-free DNA may be potentially useful for evaluation of response to treatment. The patient with WDLPS and patients with other soft tissue tumors in differential diagnosis were negative for the *MDM2* amplification in cell-free DNA. In summary, we demonstrate the feasibility of detecting amplification of *MDM2* and other DDLPS-associated genes in plasma cell-free DNA using technology that is already routinely applied for other clinical indications. Our results may have clinical implications for improved diagnosis and surveillance of patients with retroperitoneal tumors.

## Introduction

Non-invasive sampling of tumor-derived genetic material through liquid biopsies may be beneficial for an improved management of patients with soft tissue tumors. Liquid biopsies present major advantages compared to traditional tissue biopsies, as the blood draw for liquid biopsy is less invasive for the patient and the analysis of circulating tumor DNA (ctDNA) simultaneously integrates contributions from multiple regions of the tumor, enabling a more comprehensive capture of tumor heterogeneity [1, 2]. Profiling ctDNA has the potential to improve diagnosis, evaluation of response to treatment and long-term surveillance of patients with soft tissue tumors. Previous studies of ctDNA in patients with different types of sarcomas showed the feasibility of detecting tumor-derived genomic aberrations in circulation, but also revealed technological challenges that must be addressed to achieve sensitive detection of ctDNA. Our group and others have previously showed that tumor-derived copy number aberrations can be detected in plasma of patients with leiomyosarcoma (LMS) and leiomyoma (LM) [2–4]. In LMS patients, we also showed that the levels of ctDNA correspond with response to treatment, and that a combination approach that integrates detection of point mutations and copy number alterations substantially increases the number of molecular markers that can be tracked in plasma [2].

LMS and liposarcomas are the two most frequent types of sarcomas arising in retroperitoneum (75–85% of cases) [5]. Pre-operative diagnosis of these tumors relies mostly on imaging and biopsy, but accurate distinction between these entities may be challenging. We sought to evaluate whether detection of tumor-type specific aberrations in plasma of patients with well-differentiated and dedifferentiated liposarcomas (WDLPS/DDLPS) may help address this clinical challenge. WDLPS and DDLPS frequently harbor supernumerary ring chromosomes with amplification of the 12q13–15 region [6–8]. This aberration results in amplification of *MDM2*, *CDK4* and *HMGA2* genes that serve as highly specific markers of these tumors [7]. DDLPS also harbor recurrent amplifications of genes that are implicated in the inhibition of adipocytic differentiation, such as *ASK1 (MAP3K5)* on 6q23 and *JUN* on 1p32 [6, 7]. Given the almost universal amplification of *MDM2* in WDLPS and DDLPS, detection of this genomic aberration in ctDNA could be clinically useful for non-invasive distinction between these liposarcomas and other retroperitoneal tumors in the appropriate clinical context. The feasibility of detecting *MDM2* amplification in circulation has been previously addressed in two independent studies. Braig et al. demonstrated in 5 patients with WDLPS/DDLPS that a digital droplet PCR assay is not sensitive enough to detect *MDM2* amplification in plasma cell-free DNA [9]. In another study, Casadei et al. performed real-time PCR analysis of DNA extracted from extracellular vesicles from serum of 16 patients with DDLPS, and 6 healthy donors [10]. The results of Casadei et al. showed that patients with DDLPS as a group have a higher number of *MDM2* cell-free DNA molecules in comparison to healthy donors, but the method applied in this study did not allow to identify specific patients with *MDM2* amplification in the circulation. The analytical approach applied in that study is difficult to perform in a standardized manner, which taken together with a relatively laborious process of extraction and quality control of the extracellular vesicles, may not be practical for a direct implementation in the clinic. In the present study, we explored the feasibility of detecting *MDM2* amplification in cell-free DNA of patients with WDLPS and DDLPS by applying shallow whole genome sequencing, which is a well-established method for profiling copy number alterations in DNA extracted from tissues, blood and plasma. This technique is routinely used for detection of fetal chromosomal abnormalities in prenatal testing [11], and has also been successfully applied for the assessment of genome-wide copy number alterations in cell-free DNA of patients with multiple types of tumors [2, 3, 12, 13]. Here, in a limited number of patients, we demonstrate the feasibility of detecting amplification of *MDM2* and other DDLPS-associated genes by shallow whole genome sequencing of cell-free DNA.

## Materials and methods

### Patient cohort

We analyzed plasma specimens obtained from three patients with DDLPS and one patient with WDLPS. WDLPS and DDLPS tumors were diagnosed following the standard surgical pathology criteria. WDLPS tumors were hypocellular and consisted of scattered fibrous bands and large atypical cells. DDLPS were highly cellular with frequent mitotic figures, and had a well-differentiated component.

In addition, we obtained plasma samples from 11 patients with other soft tissue tumors in differential diagnosis: 1 patient with lipoma, 1 patient with pleomorphic liposarcoma, 1 patient with undifferentiated sarcoma lacking *MDM2* amplification with a history of WDLPS, and 8 patients with LMS. Seven of the LMS patients were described in our previous LMS-focused study [2].

All patients enrolled in this study were treated at the Stanford Cancer Institute and provided informed written consent to participate in the study, donate tumor and blood specimens, and have their medical records used for research. The study was approved by the Stanford University Institutional Review Board (IRB-31067).

### Molecular characterization of tumor specimens

Interphase fluorescence in situ hybridization (FISH) was performed on archival paraffin-embedded tumor specimens using the Zyto*Light* SPEC MDM2/CEN 12 Dual Color Probe (ZytoVision GmbH; Bremerhaven, Germany), which identifies amplification of the *MDM2* gene on chromosome 12 and includes a chromosome 12 centromeric control probe (D12Z3). Unequivocal amplification is defined as innumerable *MDM2* signals (>10 signals per cell) and an *MDM2*:control ratio greater than 2 in at least 25 nuclei. The absence of *MDM2* amplification was evaluated based on the analysis of 100 nuclei. FISH was performed on all three DDLPS and the cases of lipoma, pleomorphic sarcoma, undifferentiated sarcoma and one high-grade LMS. One LMS patient in our cohort (LMS8) demonstrated loss of expression of selected myogenic markers over time. This tumor was tested for *MDM2* amplification with a negative result. In the remaining 7 LMS cases, the status of *MDM2* locus was evaluated based on the previously published data from genome-wide copy number profiling using OncoScan FFPE Assay (Affymetrix, Santa Clara, CA) [2].

### Blood sample collection and processing

Blood samples were collected into EDTA tubes (Beckton Dickinson) or Cell-free DNA BCT tubes (Streck). Plasma was separated by centrifugation at 2,500g for 20 minutes at room temperature and stored at -80°C.

Blood specimens from 428 healthy volunteers (214 females and 214 males) were collected into Cell-free DNA BCT tubes (Streck) for a study at the Catholic University of Leuven. Collection of plasma from these asymptomatic donors was approved by the local Institutional Review Board. All volunteers were 65 years or older.

### Copy number profiling in cell free DNA from plasma specimens

Cell-free DNA was extracted from 3mL of plasma using the QIAamp Circulating Nucleic Acid Kit (Qiagen) and half of the yield was used for the library construction. Sequencing libraries were prepared with the TruSeq Chip preparation kit (Illumina), indexed, and pooled for multiplex sequencing on Illumina HiSeq2500. Sequencing was performed in a 1x50bp mode or a 1x36bp mode, and at least 10 million reads per sample were required from the plasma specimens.

Sequencing reads were mapped to the GRCh37/hg19 reference genome using BWA-MEM with the default settings (version 0.7.10) [14]. The pseudo-autosomal region on chromosome Y was masked in the reference genome. Duplicate reads were removed using SAMtools (version 0.1.18) [15]. A summary of mapping and deduplication statistics, and the estimated genome-wide coverage are included in S1 Table. Copy number variants in cfDNA were identified using the depth-of-coverage Plasma-Seq algorithm [16]. We used Plasma-Seq version 0.6 that uses DNAcopy from Bioconductor and does not require the CGHweb package. The analysis was performed as described previously with the following modifications: 1) sequencing reads of 50bp or 36bp were used instead of 150bp; 2) genome was divided into 100,000 windows instead of 50,000, where each window contains the same amount of mappable reads; 3) because of the increased number of windows, the average length of the bins was only 28kb and not 56kb; 4) data from 189 female and 189 male healthy donors were used as the non-tumor controls in contrast to the 10 female and 10 male control samples included originally (as described previously) [2]. Sequencing reads from the samples sequenced in 1x50bp mode were trimmed before processing to the length of 36bp. We first characterized the variability of the read counts in a reference set of 378 healthy donors (189 males and 189 females) to calibrate the Plasma-Seq algorithm. An increase or decrease in the number of normalized sequencing reads was expressed as a Z-score. Next, we applied Plasma-Seq algorithm with these settings to an independent group of 50 healthy donors (as described previously) [2], to define the genome-wide segmented Z-scores for different sizes of genomic regions that set the specificity at 98% (allowing only 1/50 healthy donors to carry a copy number alteration of a certain size in cfDNA). We defined segmented Z-scores of < -5.69 and > 5.69 as significantly under- and overrepresented regions, respectively.

All raw sequencing files are available in the NCBI Sequence Read Archive (SRA) database (accession number PRJNA754199).

## Results

### Detection of *MDM2* amplification in tumor specimens

**Fig 1:**
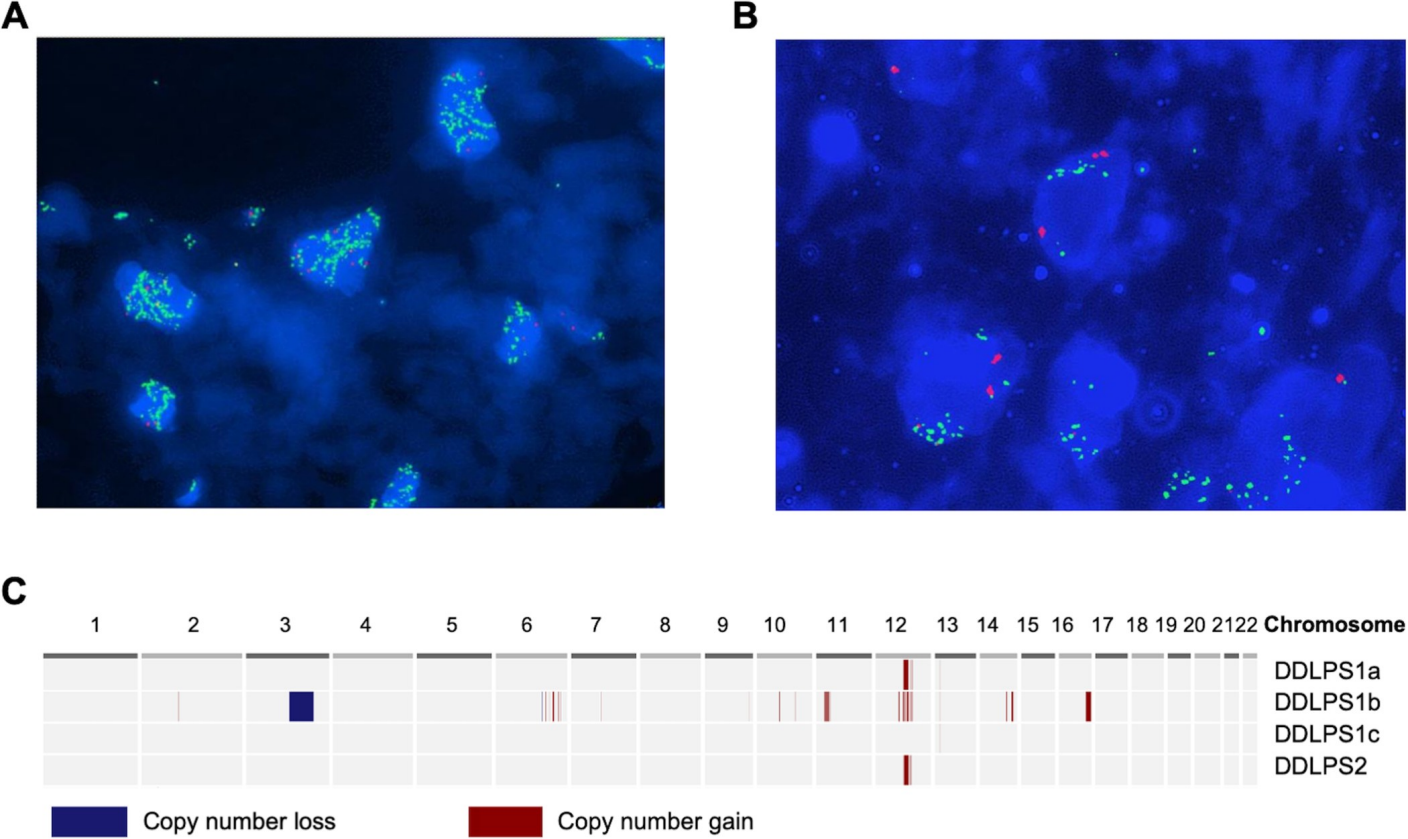
*MDM2* amplification in tumor specimens and genome-wide DNA copy number profiles of plasma specimens of two patients with DDLPS. A) and B) Detection of high-level amplification of *MDM2* in tumor specimens from patients DDLPS1 and DDLPS2, respectively. Green fluorescent signal–ZyGreen labeled polynucleotides, which target sequences at 12q15 harboring the *MDM2* gene region. Red fluorescent signal–ZyOrange labeled polynucleotides, which target sequences at 12p11.1-q11 specific for the alpha satellite centromeric region D12Z3 of chromosome 12. C) Copy number alterations detected in autosomal cell-free DNA of patients DDLPS1 and DDLPS2. The *MDM2* gene is in the 12q15 locus.

All three patients with DDLPS had diagnostic FISH performed on the primary tumor specimens, which showed that all 3 tumors carried *MDM2* amplification (Table 1) (representative FISH images from patients DDLPS1 and DDLPS2 are shown in Fig 1A and 1B). All DDLPS contained areas with lipomatous differentiation. The patient with WDLPS did not have FISH done on the primary or recurrent tumor specimens, but showed a classic histology and protein expression of p16 by immunohistochemistry. *MDM2* amplification was not detected by FISH in the tumors of patients with lipoma, pleomorphic LPS, undifferentiated sarcoma and one LMS patient. Tumor material from the remaining 7 LMS patients had previously been studied by SNP arrays (a median of 3 tumors per patient) and none of these specimens harbored amplification of the *MDM2* locus [2]. Clinical features of the DDLPS and WDLPS patients are summarized in Table 1.

**Table 1:** Summary of clinical features of DDLPS and WDLPS patients.

No.	Patient ID	Diagnosis	Sex	Age at diagnosis [years]	Site of primary tumor	Size of primary tumor [cm]	*MDM2* FISH	# of plasma samples analyzed
1	DDLPS1	DDLPS	F	64	Abdomen	19	*MDM2* amp	3
2	DDLPS2	DDLPS	M	66	Thigh	25	*MDM2* amp	1
3	DDLPS3	DDLPS	F	54	Retroperitoneum	14	*MDM2* amp	2
4	WDLPS1	WDLPS	M	60	Retroperitoneum	16	not done	4

Interestingly, patient DDLPS1 was initially diagnosed with LMS based on a core biopsy from the retroperitoneal mass, which showed a spindle neoplasm that expressed smooth muscle markers. However, eight months after the initial diagnosis, this tumor was re-classified as DDLPS based on examination of the surgically excised tumor that showed focal well-differentiated LPS and the presence of *MDM2* amplification by FISH analysis (Fig 1A). This case illustrates a common problem in the differential diagnosis of retroperitoneal soft tissue tumors.

### Detection of *MDM2* amplification and other DDLPS-specific copy number alterations in plasma specimens

We performed shallow whole genome sequencing of cell-free DNA extracted from 10 plasma samples from 4 patients with DDLPS/WDLPS, and 31 plasma samples from 11 patients with other types of soft tissue tumors. The median genome-wide coverage of sequencing across all 43 plasma samples was 0.15x (S1 Table). Table 2 shows the Z-score values in the genomic region overlapping with *MDM2* locus in all 41 plasma samples.

**Table 2:** Detection of *MDM2* amplification in cell-free DNA of patients with different types of soft tissue tumors.

No.	Plasma sample ID	Z-score in *MDM2* locus	Estimated genome-wide coverage	*MDM2* amplification in cell-free DNA	Tumor present at the time of blood collection
1	DDLPS_1a	8.9	0.17x	Detected	Yes
2	DDLPS_1b	90.7	0.11x	Detected	Yes
3	DDLPS_1c	0.2	0.16x	Not detected	No
4	DDLPS_2	13.8	0.09x	Detected	Yes
5	DDLPS_3a	1.0	0.22x	Not detected	Yes
6	DDLPS_3b	0.2	0.48x	Not detected	No
7	WDLPS_1a	0.4	0.2x	Not detected	Yes
8	WDLPS_1b	1.5	0.23x	Not detected	Yes
9	WDLPS_1c	0.6	0.27x	Not detected	Yes
10	WDLPS_1d	0.8	0.21x	Not detected	Yes
11	Pleomorphic LPS	0.2	0.16x	Not detected	Yes
12	LP	0.2	0.12x	Not detected	Yes
13	UPS	1.3	0.4x	Not detected	Yes
14	LMS1a	0.0	0.17x	Not detected	Yes
15	LMS1b	-0.6	0.17x	Not detected	Yes
16	LMS2a	0.5	0.13x	Not detected	Yes
17	LMS2b	0.7	0.14x	Not detected	Yes
18	LMS2c	-0.3	0.14x	Not detected	Yes
19	LMS3a	-5.7	0.14x	Not detected	Yes
20	LMS3b	0.1	0.15x	Not detected	Yes
21	LMS3c	-5.1	0.15x	Not detected	Yes
22	LMS3d	-3.4	0.13x	Not detected	Yes
23	LMS3e	-2.0	0.15x	Not detected	Yes
24	LMS4a	-0.4	0.15x	Not detected	Yes
25	LMS4b	0.1	0.15x	Not detected	Yes
26	LMS4c	-0.9	0.13x	Not detected	Yes
27	LMS4d	-3.0	0.13x	Not detected	Yes
28	LMS4e	-0.1	0.14x	Not detected	Yes
29	LMS4f	1.4	0.15x	Not detected	Yes
30	LMS5a	-3.0	0.13x	Not detected	Yes
31	LMS5b	-0.5	0.13x	Not detected	Yes
32	LMS5c	-0.3	0.16x	Not detected	Yes
33	LMS5d	-3.4	0.15x	Not detected	Yes
34	LMS5e	-2.3	0.15x	Not detected	Yes
35	LMS6a	0.4	0.16x	Not detected	Yes
36	LMS6b	1.5	0.14x	Not detected	Yes
37	LMS7a	-0.2	0.2x	Not detected	Yes
38	LMS7b	-0.5	0.19x	Not detected	Yes
39	LMS7c	-1.5	0.18x	Not detected	Yes
40	LMS7d	-0.1	0.14x	Not detected	Yes
41	LMS8	1.3	0.11x	Not detected	Yes

**Fig 2:**
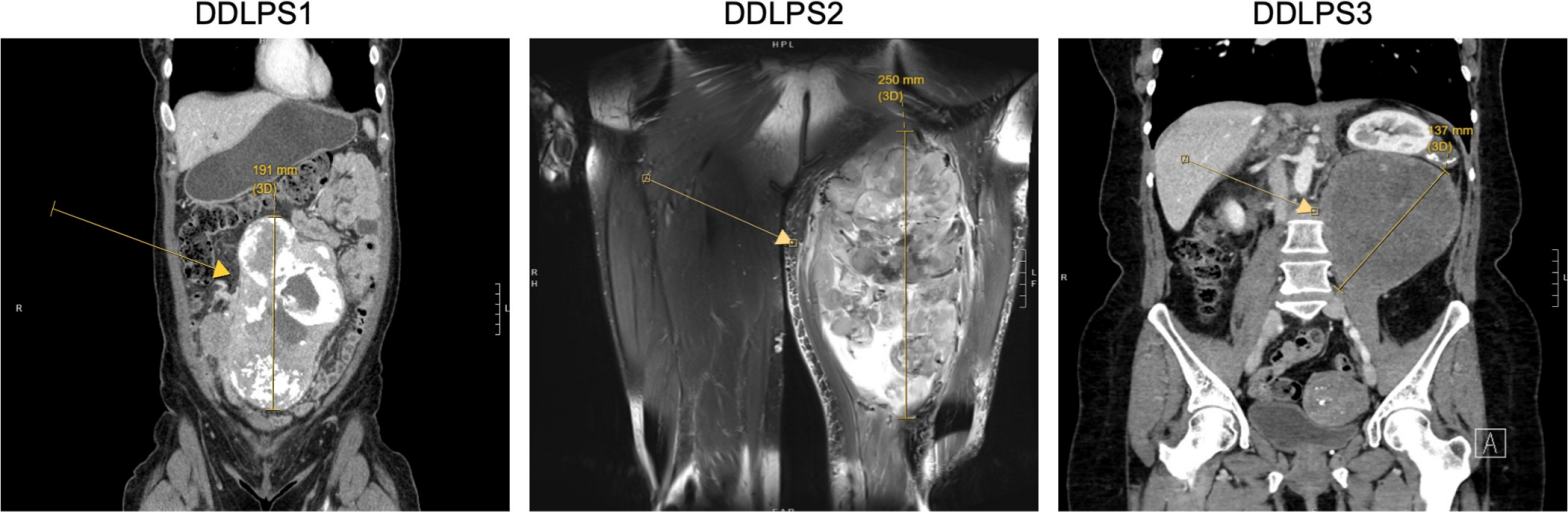
Computed tomography scans (DDLPS1 and DDLPS3) and a T2 fat-suppressed magnetic resonance image (DDLPS2) of patients with DDLPS. The tumor of patient DDLPS3 was mostly well differentiated liposarcoma, which is reflected on imaging by the low density, homogenous appearance.

Two of three (66%) patients with DDLPS patients had *MDM2* amplification present in cell-free DNA (Fig 1C). The DDLPS patient with undetectable amplification of *MDM2* in cell-free DNA had the smallest tumor size (14cm) compared to the two patients with detectable amplification (19cm and 25cm) (Table 1). Furthermore, the imaging characteristics of this 14cm tumor (DDLPS3) showed much more features of adipose tissue compared to the other two tumors (Fig 2). Pathologic examination after surgical resection showed the 14cm mass to be mostly composed of WDLPS with only a 1.7cm focus of DDLPS. In contrast, we did not detect *MDM2* amplification in cell-free DNA of any of four plasma samples of a patient with WDLPS.

Patient DDLPS1 had *MDM2* amplification detectable in 2 of 3 longitudinal plasma samples (Fig 1C). In addition, our genome-wide approach allowed us to identify amplifications in cell-free DNA spanning *ASK1* (*MAP3K5*) gene in 6q23 locus and *GATA1* gene in Xp11.23 locus in cell-free DNA extracted from the second plasma specimen from this patient (Table 3 and Fig 1C). These two genes are known to be frequently amplified in DDLPS tumors.

**Table 3:** Copy number alterations identified in cell-free DNA of patients DDLPS1 and DDLPS2 that affect genes known to be recurrently amplified in DDLPS.

Plasma sample ID	Cytoband	Genes amplified in cell-free DNA
DDLPS1_a	12q13-21	*MDM2*, *CPM*, *NAV3*
DDLPS1_b	12q13-21	*LRP1*, *GLI1*, *DDIT3*, *TSPAN31*, *CDK4*, *HMGA2*, *MDM2*, *CPM*, *YEATS4*, *FRS2*, *NAV3*, *PTPRQ*
DDLPS1_b	6q23	*ASK1 (MAP3K5)*
DDLPS1_b	Xp11.23	*GATA1*
DDLPS2	12q13-21	*MDM2*, *CPM*, *YEATS4*, *FRS2*, *NAV3*, *PTPRQ*

Successful detection of *MDM2* amplification in cell-free DNA did not appear to be associated with the depth of sequencing. The median sequencing coverage of cell-free DNA extracted from the 3 plasma samples positive for *MDM2* amplification was 0.11x, which is below the median coverage across all samples analyzed in this study. While the number of patients in our study was low, the results indicate that ctDNA was more likely to be detected in patients with larger tumors that have a significant proportion of DDLPS histology, and may be more likely to be detected in patients with DDLPS, which are usually highly cellular tumors, as compared to WDLPS that are hypocellular tumors. However, the feasibility of detection of *MDM2* amplification in plasma cell-free DNA of patients with WDLPS warrants further studies.

We achieved 100% specificity of this analysis, as *MDM2* amplification was not detected in plasma specimens of 11 patients with other tumor types that are considered in differential diagnosis of WDLPS/DDLPS (median Z-score of -0.2, range: -5.7 to 1.3). Also, *MDM2* amplification was not detected in plasma cell-free DNA of any of the 50 healthy donors (median Z-score of 0.1, range: -3.9 to 2.5).

### Levels of *MDM2* amplification in serial plasma samples from a DDLPS patient correspond with response to treatment

To assess whether the levels of *MDM2* in cell-free DNA correlate with response to treatment, we analyzed three longitudinal blood specimens from one patient with DDLPS. Patient DDLPS1 was a 63-year-old female who presented with a dominant 19x15x10cm intra-abdominal mass and separate masses involving the left hemidiaphragm, kidney, and the periaortic and retrocaval areas. A core biopsy of the abdominal mass showed a tumor consisting of spindle cells that expressed desmin and caldesmon with focal SMA staining. A diagnosis of LMS was initially made and the patient started neoadjuvant chemotherapy with gemcitabine and docetaxel. The patient did not respond to this treatment, nor to a subsequent therapy with doxorubicin. Next, the patient was treated with ifosfamide and radiation followed by radical resection of all tumor masses where focal areas of WDLPS were noted adjacent to the spindle cell tumor. A FISH test performed on the dedifferentiated component of the tumor that was surgically removed 8 months after the initial diagnosis was positive for *MDM2* amplification.

**Fig 3:**
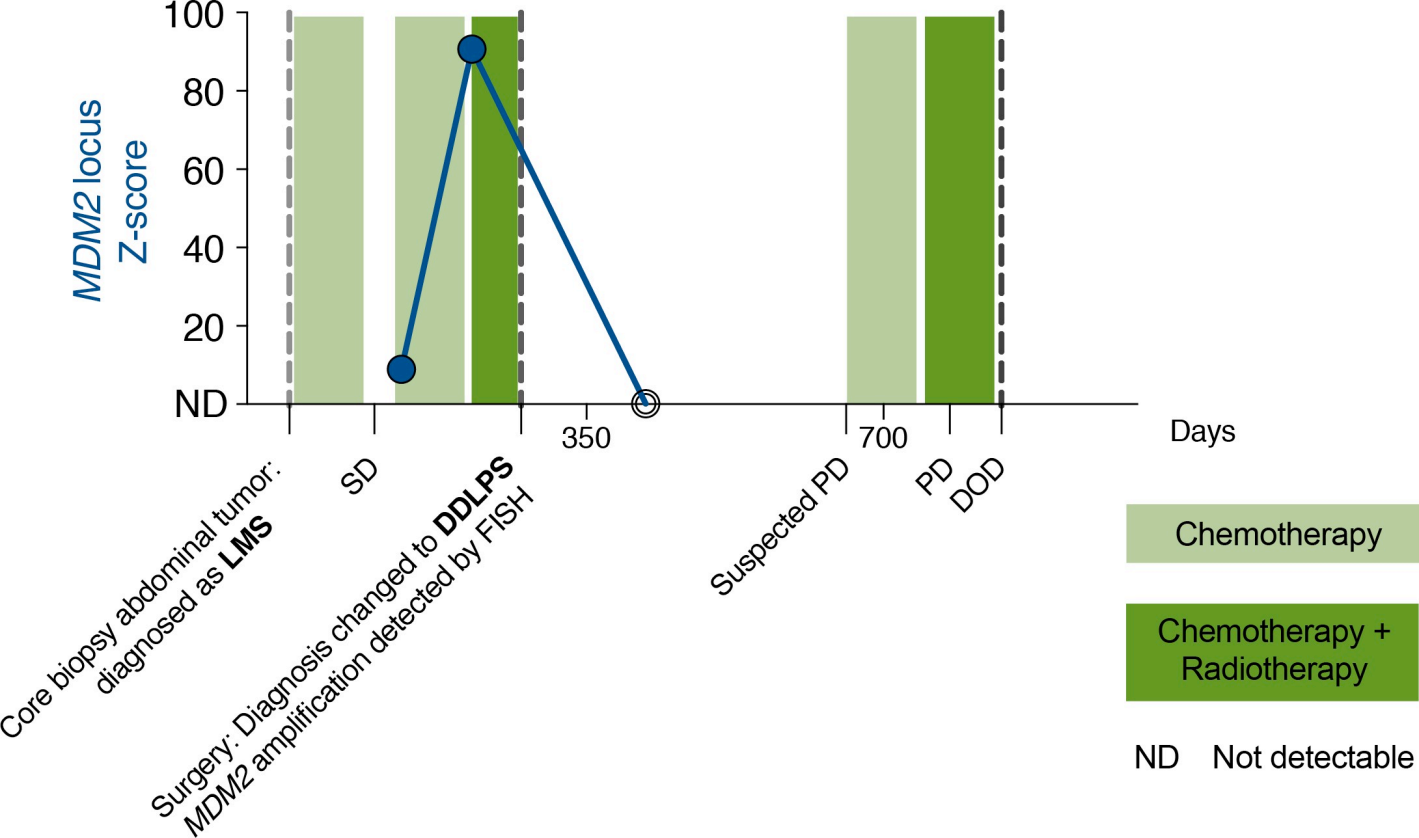
Longitudinal monitoring of *MDM2* amplification in cell-free DNA of patient DDLPS1. [SD–stable disease; PD–progressive disease, DOD–died of disease, ND–not detectable].

Fig 3 depicts how ctDNA levels (expressed as the Z-score in the *MDM2* locus) correlated with response to treatment in this patient. The first two blood samples were obtained 3 and 6 months after the initial diagnosis in a period when the patient was progressing during chemotherapy. The first blood sample showed *MDM2* amplification with a Z-score of 8.9 and in the second sample we observed an increased Z-score of 90.7. In the third blood sample, collected 5 months after resection of the primary mass, *MDM2* amplification became undetectable in cell-free DNA (Z-score = 0.2). At the time of the third blood collection, the patient had only small stable soft tissue nodules (not larger than 2.4 cm). A CT scan taken 13 months after the surgery showed progression of disease. The patient did not respond to subsequent treatment regimens and passed away 27 months after the initial diagnosis.

## Discussion

In this study we demonstrate that *MDM2* amplification can be detected by shallow whole genome sequencing of plasma cell-free DNA from patients with DDLPS. We show the feasibility of this approach by applying a protocol that is already routinely used in the clinical setting for non-invasive prenatal testing, indicating that the same technology may be useful for the management of patients with DDLPS. We also show that the presence of *MDM2* amplification in ctDNA may be associated with response to treatment. This non-invasive approach may help address the clinical challenges related to diagnosis, treatment and follow-up of these tumors.

Liposarcomas and LMS are the two most frequent types of sarcomas arising in retroperitoneum (75–85% of cases) [5]. Pre-operative diagnosis of these entities relies mostly on imaging and evaluation of tumor biopsy. If confirmed in a larger series, our data suggest that an ancillary non-invasive test for amplification of *MDM2* and other DDLPS-associated genes in cell-free DNA may help with the pre-operative diagnosis when imaging or tumor biopsy is equivocal. In fact, a presurgical plasma test could have potentially led to an earlier re-evaluation of the biopsy-based diagnosis of LMS in patient DDLPS1 described in this study. In addition, retroperitoneal DDLPS have been shown to have a high risk for local recurrence, while having a low risk for distant metastases when compared to LMS [17]. Furthermore, a definitive pre-surgical diagnosis could help tailor neoadjuvant and adjuvant chemotherapy. The ongoing STRASS2 trial is investigating whether the use of neoadjuvant chemotherapy is beneficial in retroperitoneal sarcoma and the chemotherapy regimens differ based on the histologic diagnosis, doxorubicin/ifosfamide for DDLPS as opposed to doxorubicin/dacarbazine for leiomyosarcoma. Thus, the accurate differential diagnosis between DDLPS and LMS is important for prognostication and the choice of treatment.

Another clinical application of profiling *MDM2* amplification in plasma may be in early detection or monitoring of disease progression. As demonstrated in patient DDLPS1, the first two blood samples were positive for the presence of *MDM2* amplification, before the resection of a large primary tumor that did not respond to the neoadjuvant therapy. The third blood specimen collected 5 months after surgery was negative for *MDM2* amplification when the patient only had small stable nodules as confirmed by imaging, that were not considered to be progressive disease. These findings indicate that *MDM2* amplification may be detectable in cell-free DNA at the time of disease progression, and becomes undetectable at the time of no proven active disease. This observation in a single patient provides a rationale for a prospective study that would confirm an association between ctDNA levels and response to treatment in a larger cohort of patients with DDLPS.

Another advantage of liquid biopsy testing is that the pool of cell-free DNA in the circulation represents DNA released from different regions of the tumor, which can help overcome the challenges related to tumor heterogeneity. Retroperitoneal DDLPS present as large masses that appear heterogeneous on CT and MRI scans, and distinction from adjacent normal fat can be challenging. Microscopically, these tumors may consist of a range of different morphologies and the percentage of tumor cells with *MDM2* amplification may vary in different areas of the tumor, which may affect the proper evaluation of FISH performed on a pre-surgical biopsy. An axillary test for the presence of *MDM2* amplification in cell-free DNA may help verify the results of a traditional tumor biopsy, or may be useful if the results of tumor biopsy are equivocal or uninterpretable.

WDLPS, similar to DDLPS, harbors amplification of the *MDM2* gene but these tumors are much less cellular than DDLPS, and *MDM2* amplification can be seen in as few as 15% of the cells in WDLPS. Therefore, assuming that the ctDNA yield is proportional to tumor content, it may not be surprising that in our small series we could not detect *MDM2* amplification in patients with tumors showing primarily well-differentiated histology. Overall, it remains to be determined in a larger series whether amplification of *MDM2* and other genes in the 12q13-21 locus can be detected in cell-free DNA of patients with WDLPS. A larger study is also necessary do determine whether in patients with DDLPS the sensitivity of detection of *MDM2* amplification in plasma is associated with the extent of dedifferentiation and the depth of sequencing.

Two previous studies explored the feasibility of detecting *MDM2* amplification in the circulation. Braig et al. demonstrated that digital droplet PCR assay is not sensitive enough to detect *MDM2* amplification in cell-free DNA extracted from plasma of 5 patients with WDLPS or DDLPS [9]. Casadei et al. showed significantly increased numbers of *MDM2* DNA molecules in the extracellular vesicles extracted from serum of 16 patients with DDLPS compared to 6 healthy controls, as determined by real-time PCR [10]. However, the authors of that study described that the measurement implemented in their experiments is difficult to calibrate, which taken together with a relatively laborious process of extraction and quality control of the extracellular vesicles, may be less practical for direct implementation in the clinic. While the study of Casadei et al. demonstrated for the first time the feasibility of detecting increased number of *MDM2* DNA molecules in the circulation of patients with DDLPS as a group, the method applied in that study does not allow for the detection of *MDM2* gene amplification in individual patients. Instead, the study employed real-time PCR incorporating a standard curve methodology, using a direct comparison of patient and control samples. This approach is difficult to standardize and not practical to become broadly applied in the clinical setting. In our present study, we employed a method of ctDNA detection that is well-standardized and routinely used for prenatal screening for fetal aneuploidies. By applying shallow whole genome sequencing, we have demonstrated that it was feasible to detect *MDM2* amplification in cell-free DNA of 2/3 patients with DDLPS, and that tracking the presence of this aberration in plasma may be potentially useful for evaluation of response to treatment. Furthermore, we demonstrate that shallow whole genome sequencing of cell-free DNA allows for detecting amplification of not only *MDM2*, but also other genes that are frequently affected in DDLPS. By applying the genome-wide approach, in patient DDLPS1 we detected amplification of multiple genes in the 12q13-21 locus, *ASK1* gene in 6q23 locus and *GATA1* gene in Xp11.23 locus. ASK1 is a MAP3 kinase that inhibits adipocytic differentiation through inactivation of peroxisome proliferator-activated receptor gamma [6, 18, 19]. Amplification of *GATA1* has been reported in two independent DDLPS cases by Somaiah et al. and in 3 DDLPS tumors included in the TCGA sarcoma study [8, 20] but the specific role of this gene in DDLPS remains unknown.

In summary, we demonstrate that the liquid biopsy approach may be useful for detection of specific copy number changes in cell-free DNA of patients with DDLPS, using the same approach as is already routinely used in the clinic for non-invasive prenatal testing. We also show that this assay was highly specific for detecting *MDM2* amplification in the circulation of patients with DDLPS, as this aberration was not detectable in cell-free DNA of patients with lipoma, pleomorphic liposarcoma, undifferentiated sarcoma or LMS. The clinical implications of our findings may significantly help in accurate diagnosis, treatment and surveillance of DDLPS, but the sensitivity of this approach needs to be evaluated in a larger cohort of patients.

## Supporting information

S1 Table Summary statistics of shallow whole genome sequencing of plasma cell-free DNA. (XLSX)
